# Treatment of Developmental Dysplasia of the Acetabulum by Hip Arthroscopic Acetabuloplasty Combined With Labral Repair, Cam and Pincer Osteoplasty, Collagen‐Induced Autologous Chondroplasty, and Capsule Suture

**DOI:** 10.1002/atn2.70195

**Published:** 2026-08-04

**Authors:** Dongdong Zhou, Jin Zhang, Zhe Xue, Zhengwei Zhu, Shihao Yang, Deding Liu, Xinyue Zhang, Ceyong Sun

**Affiliations:** ^1^ Department of Sports Medical Zhengzhou Central Hospital Affiliated to Zhengzhou University Zhengzhou China; ^2^ Department of Sports Medicine Beijing Jishuitan Hospital, Capital Medical University Beijing China; ^3^ Department of Sports Injury and Arthroscopic Surgery National Institute of Sports Medicine Beijing China; ^4^ Department of Orthopaedics Huairou Hospital, Beijing Chao‐Yang Hospital, Capital Medical University Beijing China

## Abstract

Developmental hip dysplasia due to insufficient acetabular coverage and excessive pressure on the femoral head can easily lead to labral tears, damage to the labrum–cartilage junction, cam and pincer deformities, and joint capsule laxity. This may ultimately result in osteoarthritis. Here, we describe a technique that involves bone grafting with autologous bone harvested from the anterior superior iliac spine combined with labral repair, cam and pincer osteoplasty (bone resection), autologous collagen‐induced chondrogenesis, and joint capsule suture in a “five‐in‐one” procedure to treat developmental hip dysplasia.

## Graphical abstract

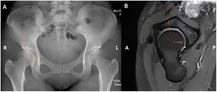

**VIDEO 1:** Treatment of Developmental Dysplasia of the Acetabulum by Hip Arthroscopic “five‐in‐one” Procedure. The patient is supine. The video shows: 1) Preoperative imaging diagnosing right hip dysplasia, labral tear, and cartilage damage; 2) Intraoperative setup with joint traction and establishment of the anterolateral, modified mid‐anterior, and distal anterolateral accessory (DALA) portals; 3) Pincer osteoplasty and suture anchor fixation for anatomical labral repair; 4) Autologous chondrogenesis induced by collagen (ACIC) via microfracture and scaffold application for cartilage defects; 5) Cam reshaping and joint capsule plication suture; 6) Autologous bone block harvesting from the anterior superior iliac spine and structural graft fixation at the acetabular roof via the DALA approach. Video content can be viewed at https://doi.org/10.1002/atn2.70195.

Developmental hip dysplasia is a common cause of hip joint pain, functional impairment, and joint instability.^1^ Due to reduced coverage of the femoral head, abnormal development of the acetabulum often leads to severe labral tears, cartilage damage, cam and pincer deformities, and laxity of the joint capsule. These may ultimately result in osteoarthritis.^2^, ^3^, ^4^

Wiberg was the first to propose using the center‐edge (CE) angle to evaluate the coverage of the femoral head by the acetabulum.^5^ Subsequently, the lateral CE angle was introduced to more accurately assess the lateral coverage of the femoral head by the acetabulum. Generally, a lateral CE angle <20° suggests hip dysplasia, whereas >25° is considered normal, although the specific values remain controversial in different studies. Ganz et al.^6^ proposed periacetabular osteotomy for the treatment of developmental hip dysplasia in adolescents and adults. Although this surgical method is effective, it is not widely accepted by patients because of its invasive nature and high technical demands. Subsequently, with the gradual development of minimally invasive techniques, Uchida et al.^7^ proposed a method for performing acetabular reorientation surgery using autogenous iliac bone grafts, under arthroscopy. This can simultaneously correct acetabular bone defects and intra‐articular injuries.

Therefore, we further optimized the technique proposed by Uchida et al. by integrating clinical practice and research. Specific improvements include the incorporation of autologous chondrogenesis induced by collagen (ACIC) and improving the use of the suture line at the tail of the anchor. Specifically, one is used for fixing the labrum and the other is used for folding and suturing the joint capsule tissue.^8^

## SURGICAL TECHNIQUE

**FIGURE 1:**
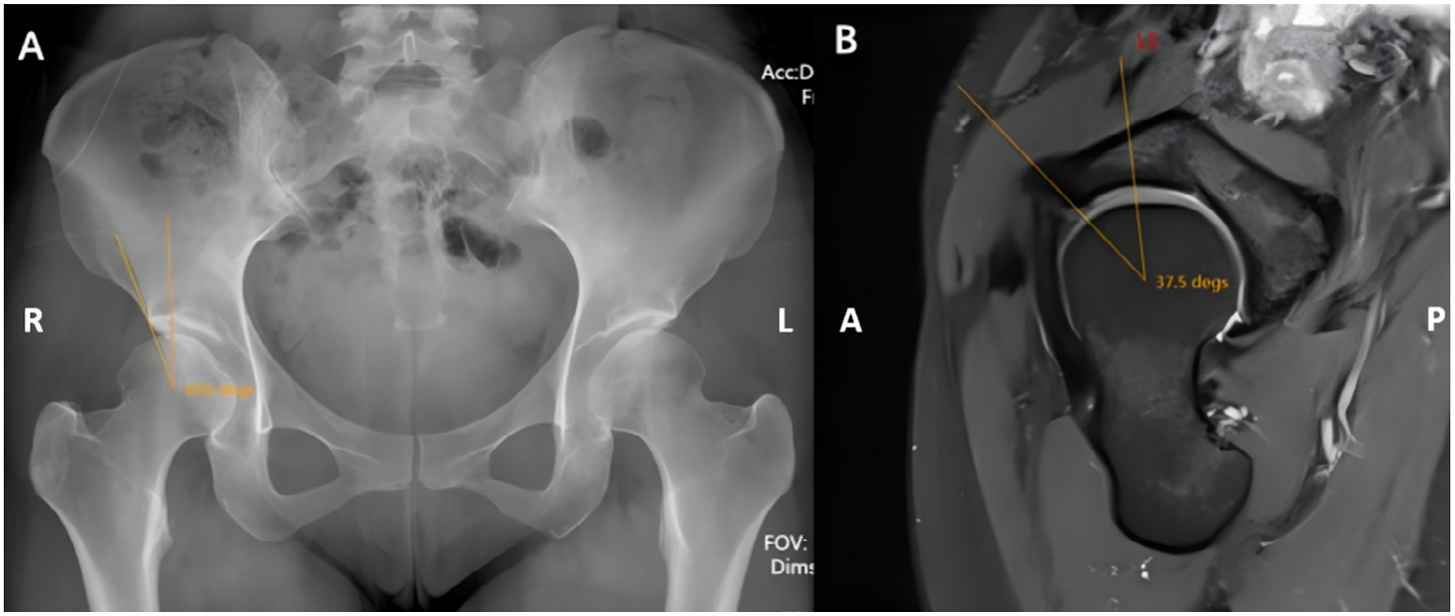
Preoperative radiographic angle measurements of the right hip. (A) Weight‐bearing radiograph measuring the lateral CE angle at 18.6°; (B) Magnetic resonance imaging sagittal view measuring the anterior CE angle at 37.5°. (A, anterior; CE, center‐edge; L, left; P, posterior; R, right.)

**FIGURE 2:**
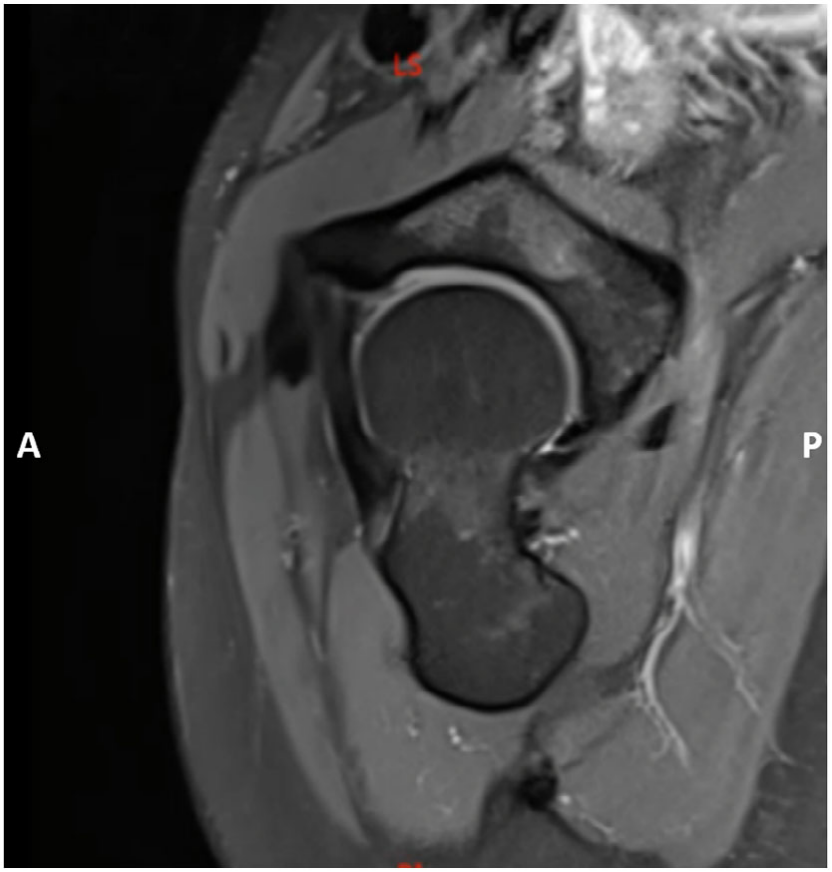
Magnetic resonance imaging (sagittal view) of the right hip joint with the patient in the supine position, showing labral damage and cartilage damage at the junction of the labrum and cartilage. (A, anterior; P, posterior.)

This technique is indicated for symptomatic adult patients presenting with persistent hip and groin pain, typically accompanied by limitations in range of motion such as flexion and rotation. Clinical indications include positive anterior impingement and FABER test results on physical examination. Radiographic indications are based on imaging confirmation of acetabular dysplasia, characterized by a deficient lateral center‐edge angle (Figure 1), along with magnetic resonance imaging evidence of a labral tear and associated cartilage damage (Figure 2).

### Diagnostic Arthroscopic Examination

**FIGURE 3:**
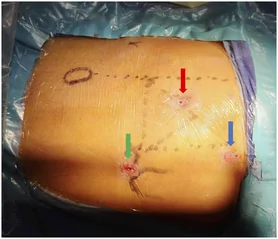
Arthroscopic surgical approaches on the right hip with the patient in the supine position: Green arrow: ALP; red arrow: MAP; blue arrow: DALA approach. (ALP, anterolateral approach; DALA, distal anterolateral accessory; MAP, modified mid‐anterior approach.)

**FIGURE 4:**
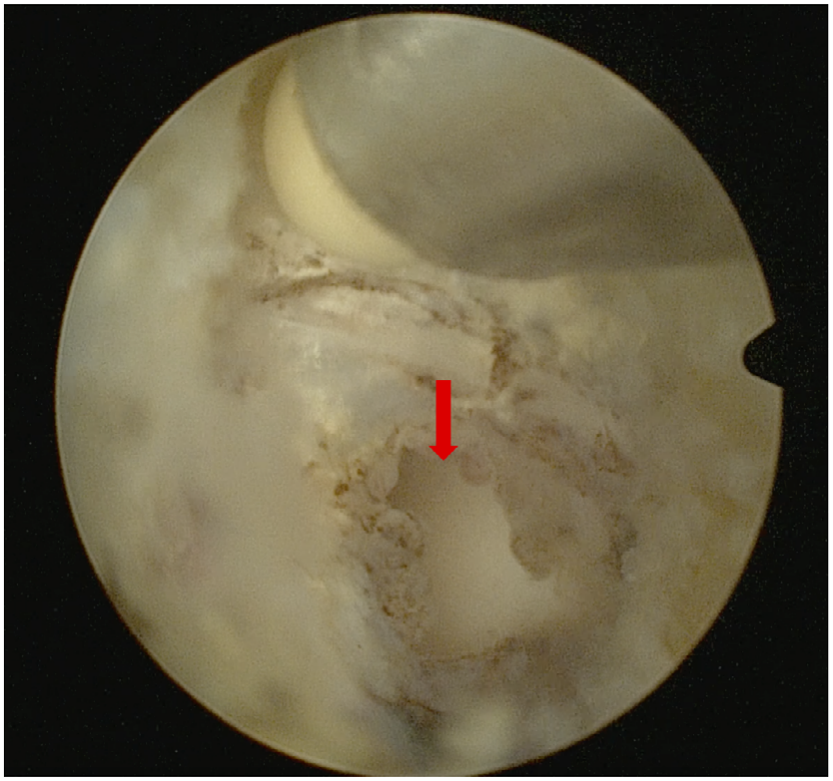
Arthroscopic view of the right hip in the supine position, viewed from the anterolateral portal. From the lateral side of the joint, entry into the joint is initiated by making a point incision on the joint capsule (red arrow) to determine the location, followed by a horizontal incision on the joint capsule.

The patient lies supine on a hip joint traction table, with a perineal post, and the right lower limb is appropriately tractioned to maintain tension. After routine disinfection of the surgical area, anterolateral, modified mid‐anterior, and distal anterolateral accessory approaches are established (Figure 3, Video 1). The synovium is cleared to expose the surface of the hip joint capsule, which is then incised at certain points (Figure 4). After assessing the current position, the joint capsule is incised transversely along the labral edge. The traction is then increased to widen the distraction between the femoral head and acetabular rim.

### Pincer Osteoplasty and Labrum Repair

**FIGURE 5:**
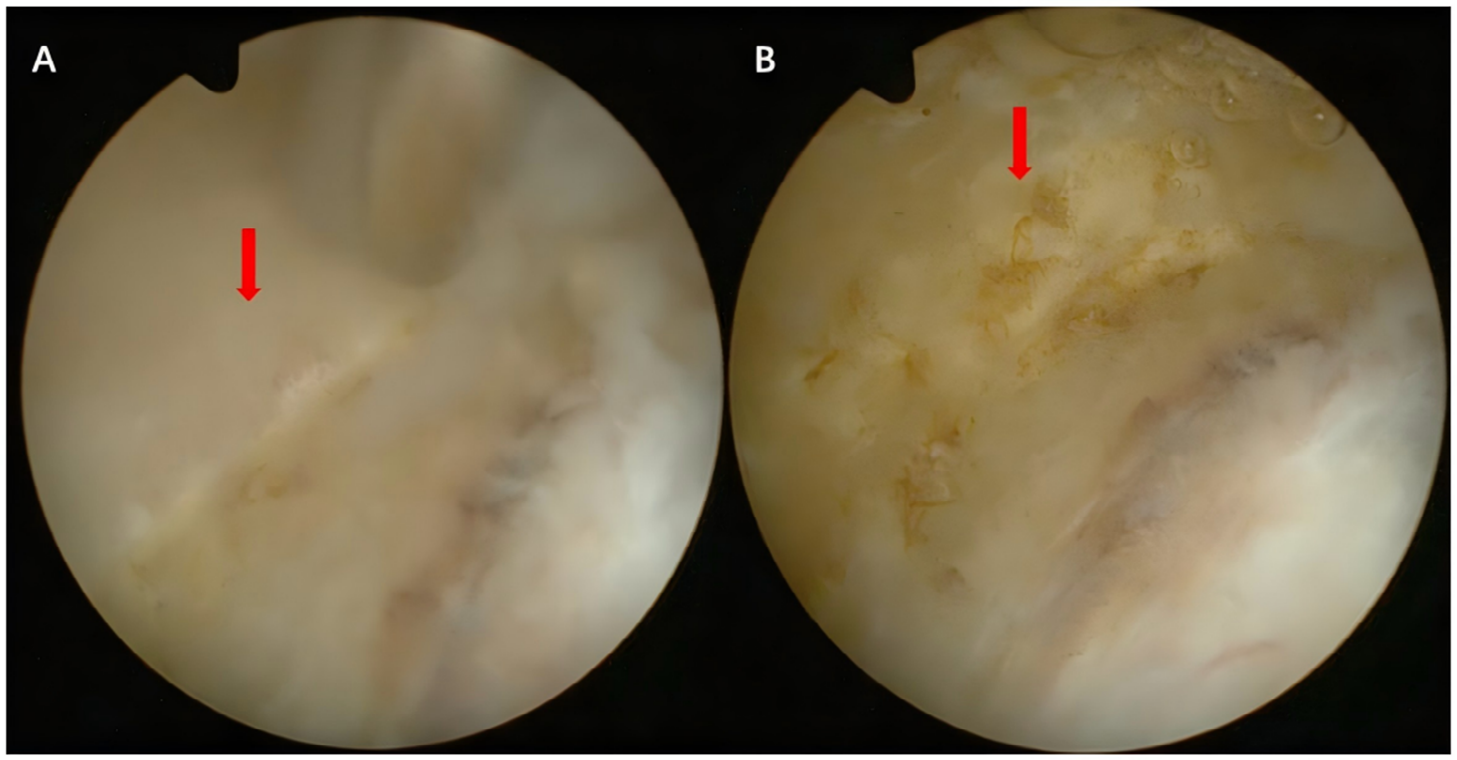
Arthroscopic view of acetabular‐side formation of the right hip in the supine position, viewed from the anterolateral portal. (A) Post‐pincer formation of the acetabular rim. (B) Prepincer formation of the acetabular rim.

**FIGURE 6:**
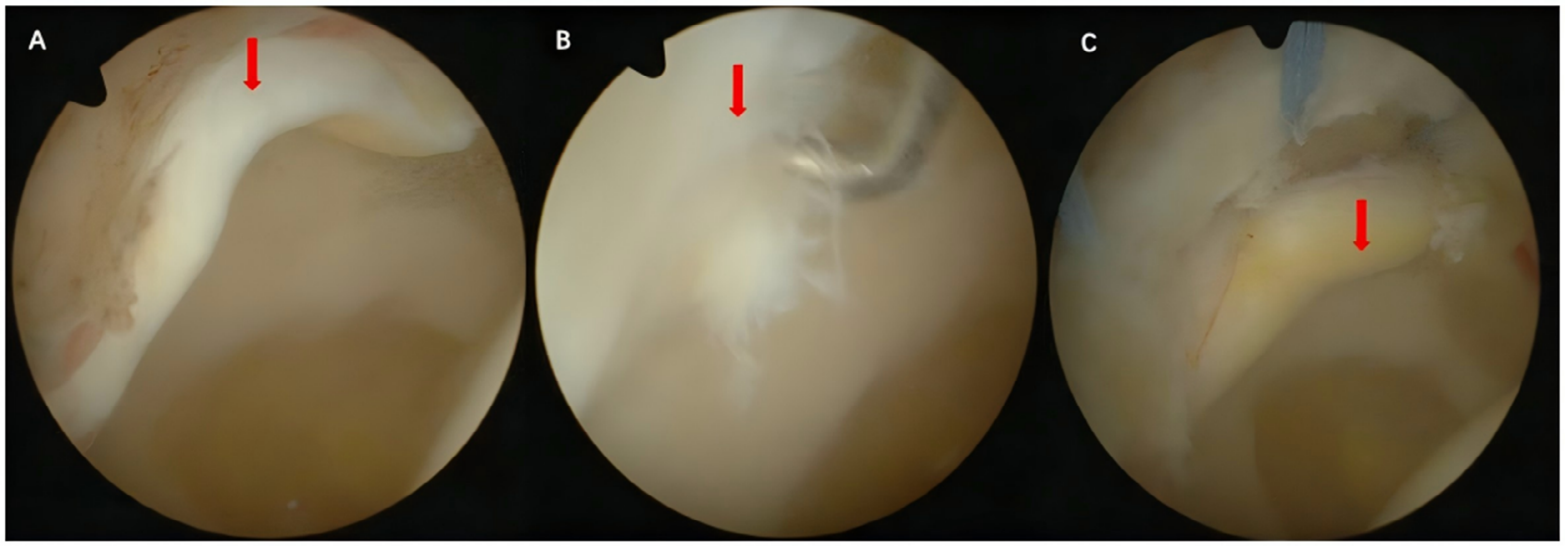
Arthroscopic view of hip joint labrum suture repair of the right hip in the supine position, viewed from the anterolateral portal. (A) Undulated configuration of the acetabular rim labrum. (B) Damage to the acetabular rim labrum. (C) Completion of labrum suture repair.

A plasma radiofrequency vaporization system (DePuy Mitek, Tokyo, Japan) is used to clear the synovial tissue at the edge of the labrum and to expose the area of acetabular overgrowth for pincer formation (Figure 5). After formation is complete, the size of the labral tear and the degree of cartilage damage at the transition zone, between the labrum and cartilage are examined. The 3 anchors (Youwin, China) are inserted in the direction of the tear. Each anchor retains 1 suture line for joint capsule suturing as a backup and then sequentially sutures, and the torn labrum is fixed using loop knots (Figure 6).

### Autologous Chondrogenesis Induced by Collagen

**FIGURE 7:**
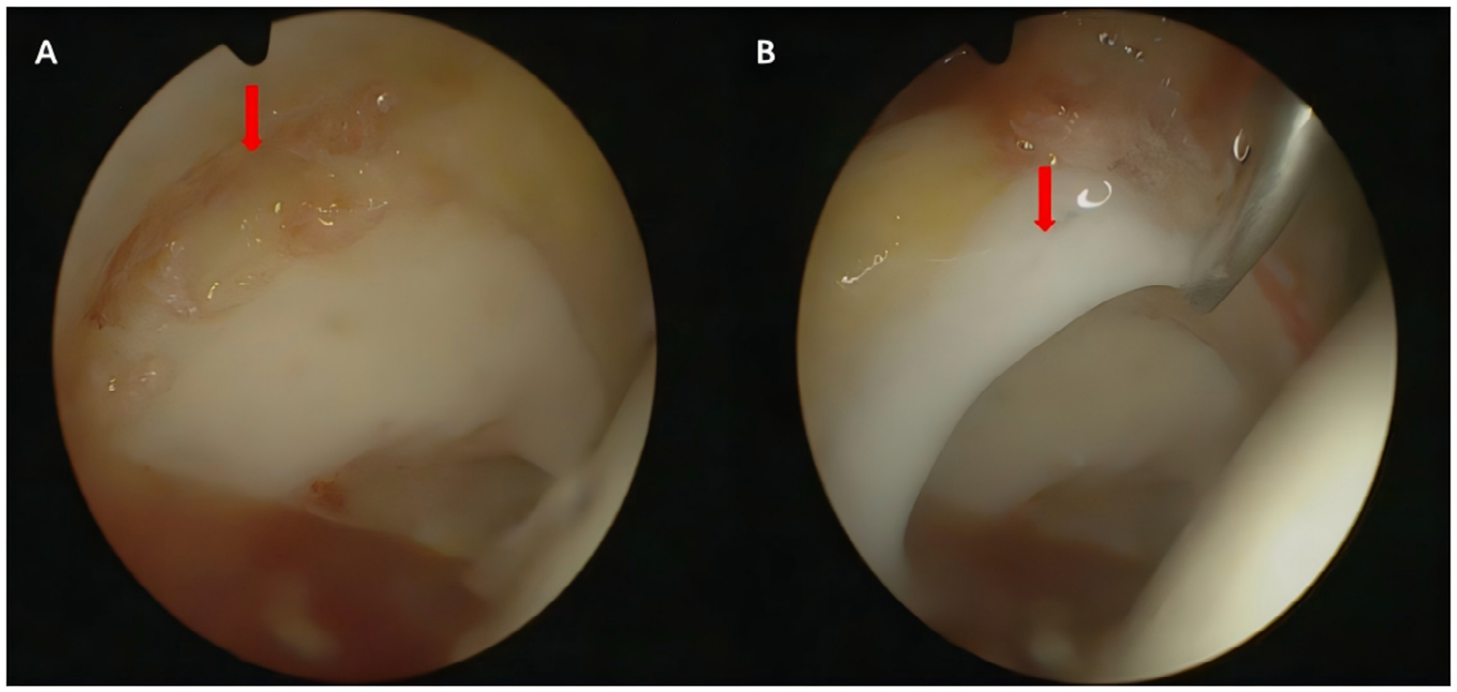
Arthroscopic view of the cartilage injury repair process of the right hip in the supine position, viewed from the anterolateral portal. (A) Cartilage injury at the junction of the labrum and cartilage. (B) A mixture of collagen cartilage repair scaffold and porcine fibrin adhesive is used to fill the area of cartilage damage.

A fluid management and tissue debridement system (DePuy Mitek, Tokyo, Japan) is used to remove the transition area of the labrum and damaged cartilage. A bone awl is used to create local microfractures, until fresh blood flows out and the irrigation fluid in the joint cavity is aspirated. A syringe containing a collagen‐based cartilage repair scaffold (COLTRIX CartiRegen) and porcine fibrin protein adhesive is prepared and is then inserted into the joint cavity through a modified anteromedial approach. The needle position is adjusted to allow for it to be located in the area of the cartilage defect and then slowly injected until the mixture completely fills the cartilage defect area (Figure 7). The syringe is maintained for 5 min and slowly withdrawn after the mixture has formed and adhered.

### Cam Reshaping and Joint Capsule Suture

**FIGURE 8:**
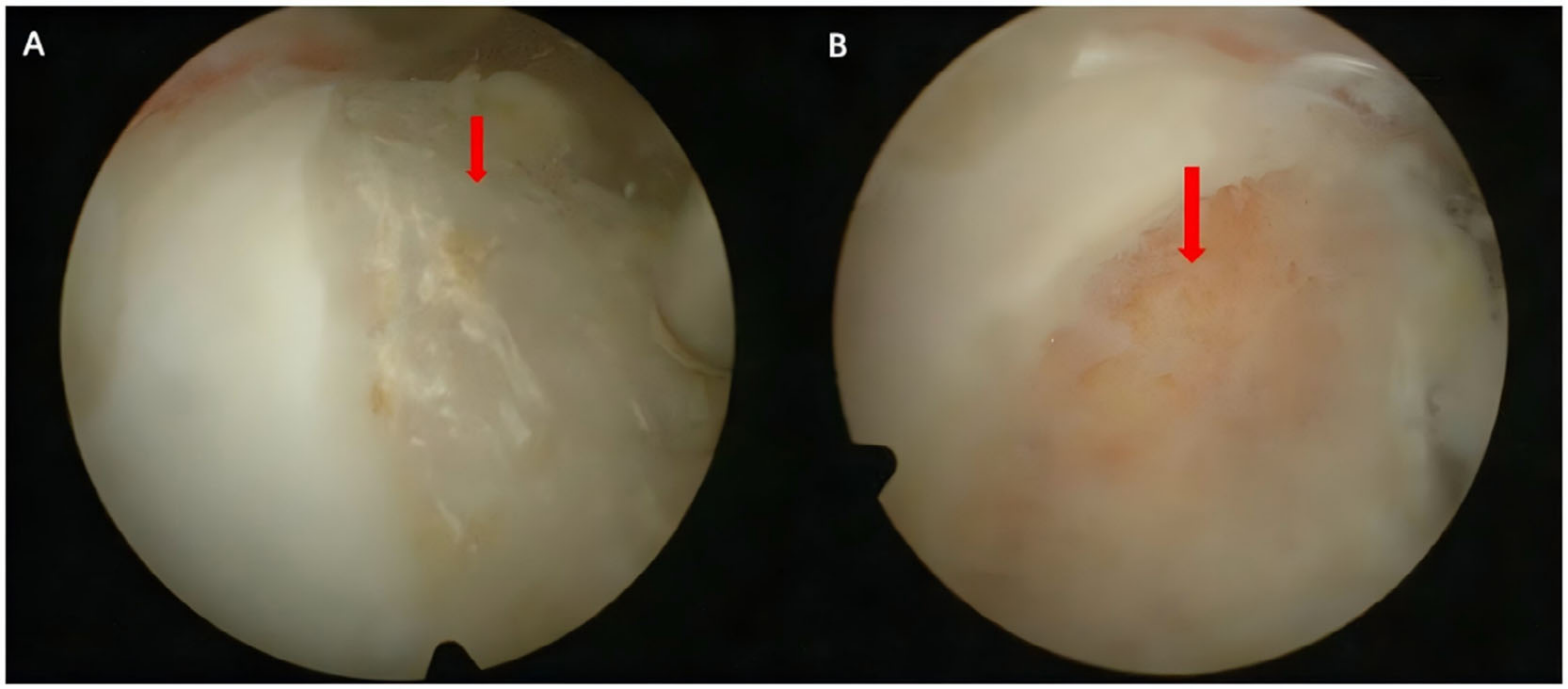
Arthroscopic view of femoral‐side reshaping of the right hip in the supine position, viewed from the anterolateral portal. (A) Preoperative femoral‐side cam reshaping. (B) Post‐operative femoral‐side cam reshaping.

**FIGURE 9:**
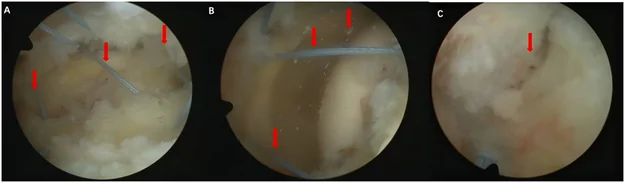
Arthroscopic view of joint capsule suturing of the right hip in the supine position. Flexion of the hip and knee to puncture the distal joint capsule with a transverse incision using a puncture clamp via both the DALA approach and the modified mid‐anterior approach, passing the proximal anchor line through the distal joint capsule. (A) Observation of the completion of passing the line on the joint capsule side via the MAP approach. (B) Observation of the completion of passing the line on the joint capsule side via the ALP approach. (C) In the extended knee and hip position, the remaining anchor line of the fixed labrum is tied and secured with the distal joint capsule suture. (ALP, anterolateral approach; DALA, distal anterolateral accessory; MAP, modified mid‐anterior.)

The femoral side cam deformity is exposed and assessed. Cam reshaping is performed using a fluid and tissue management debridement system (DePuy Mitek, Tokyo, Japan) (Figure 8). The hip and knee are flexed to allow the puncture forceps to pass through the distal anterolateral accessory and modified anterior approaches with a transverse incision made in the distal joint capsule. The proximal anchor line is then passed through the distal joint capsule. In the extended knee and hip positions, the anchor line is knotted and fixed with a suture of the distal joint capsule (Figure 9).

### Harvesting of Bone Block and Fixation of Acetabular Roof

**FIGURE 10:**
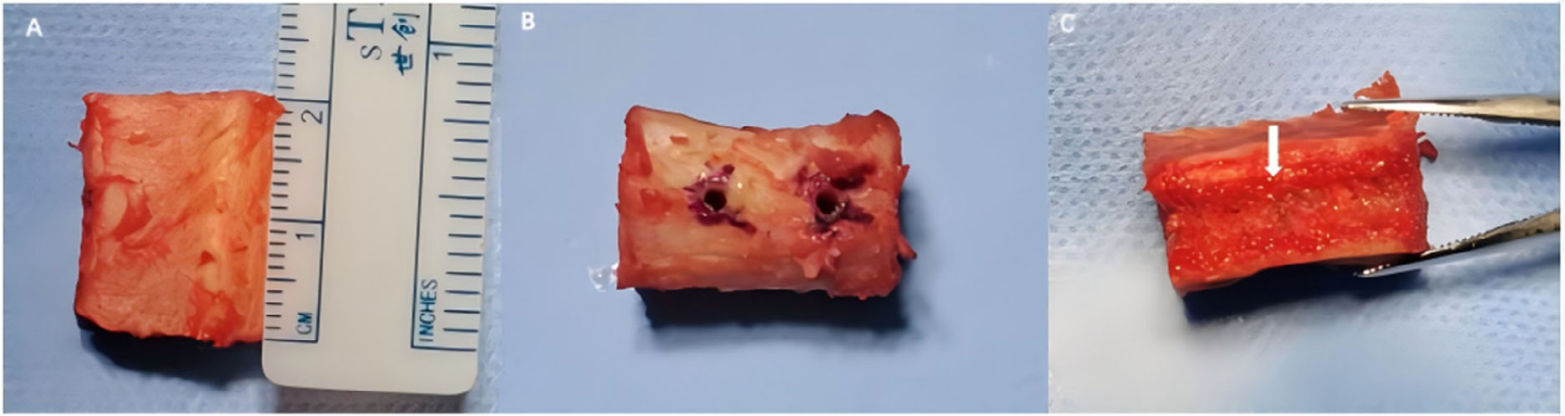
Harvesting and preparation of the anterior superior iliac spine bone block. (A) Cleaning of the tissue on the surface of the anterior superior iliac spine bone block. (B) Preparation of prefixation holes after bone harvesting from the anterior superior iliac spine. (C) Groove on the deep osteotomy surface (white arrow).

An incision is made in the right anterior superior iliac spine, perpendicular to the bone surface. The size of the bone block, particularly its width, is determined preoperatively to achieve a planned correction of the CE angle. The surgical planning is based on the principle that approximately 1 mm of lateral bone block width provides roughly 1° of CE angle increase. The target graft width is calculated accordingly to augment the deficient acetabulum to a target CE angle within the normative range (e.g., 30°‐40°), while avoiding overcoverage and iatrogenic impingement. Based on this calculation, bone blocks measuring 2 cm in length, 1 cm in width, and 1 cm in height are harvested using a bone chisel. Surface muscles and tendinous tissues are then cleaned. A 2.5‐mm hole is drilled at 5‐mm intervals on the surface of the anterior superior iliac spine for future use, and a groove is created on the deep osteotomy surface (Figure 10).

**FIGURE 11:**
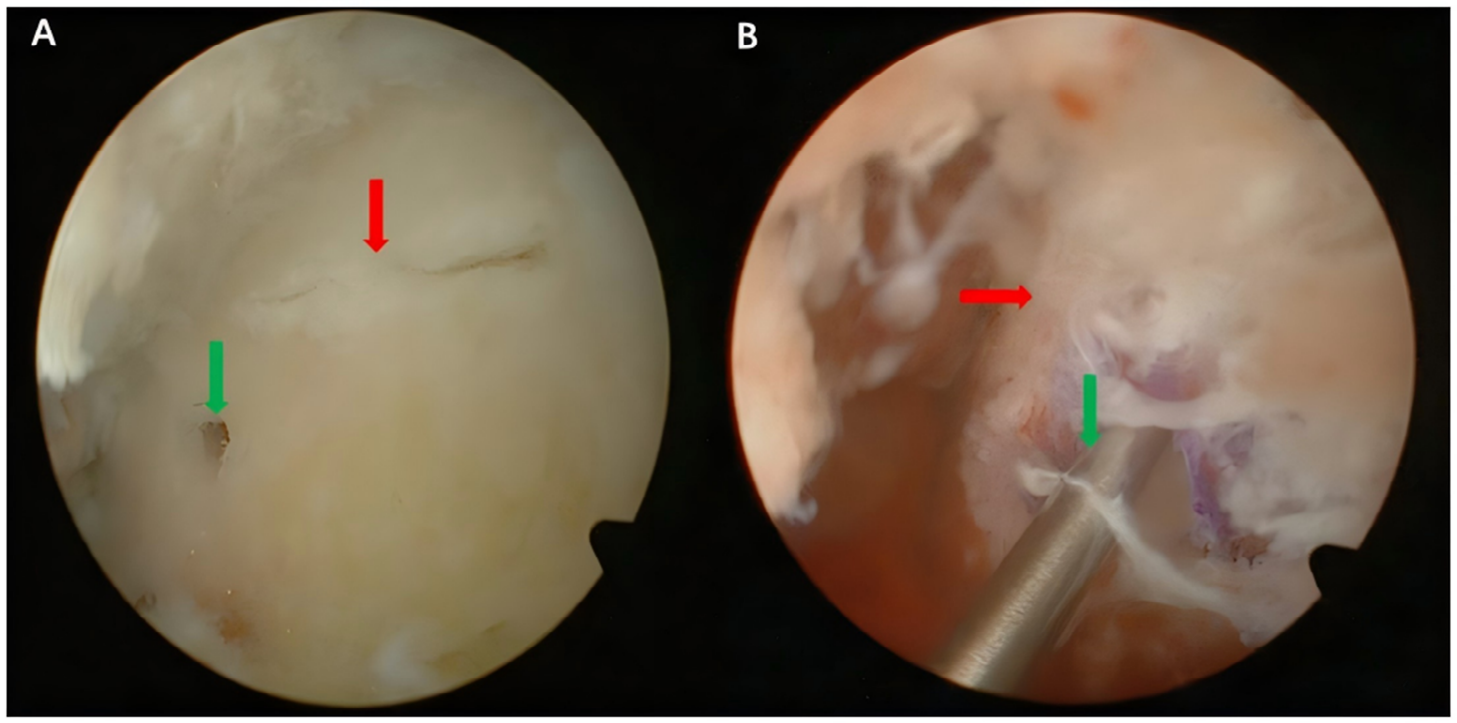
Arthroscopic view of right hip graft bed preparation and bone block insertion with the patient in the supine position. (A) Using a fluid and tissue management debridement system (DePuy Mitek, Tokyo, Japan) to clean the gap, exposing the medial edge of the acetabulum, polishing part of the cortical bone, and inserting a Kirschner wire for drilling and positioning (green arrow). The C‐arm fluoroscopy confirms the pregraft fixation position. A groove approximately 20‐mm long and 2‐mm deep is created via the distal anterolateral accessory approach (red arrow), similar to the method described by Uchida et al.^7^ (B) Insertion of the graft bone block (red arrow) along the predrilled position, allowing the bone block guide pin (green arrow) to enter the predrilled position in the acetabulum.

**FIGURE 12:**
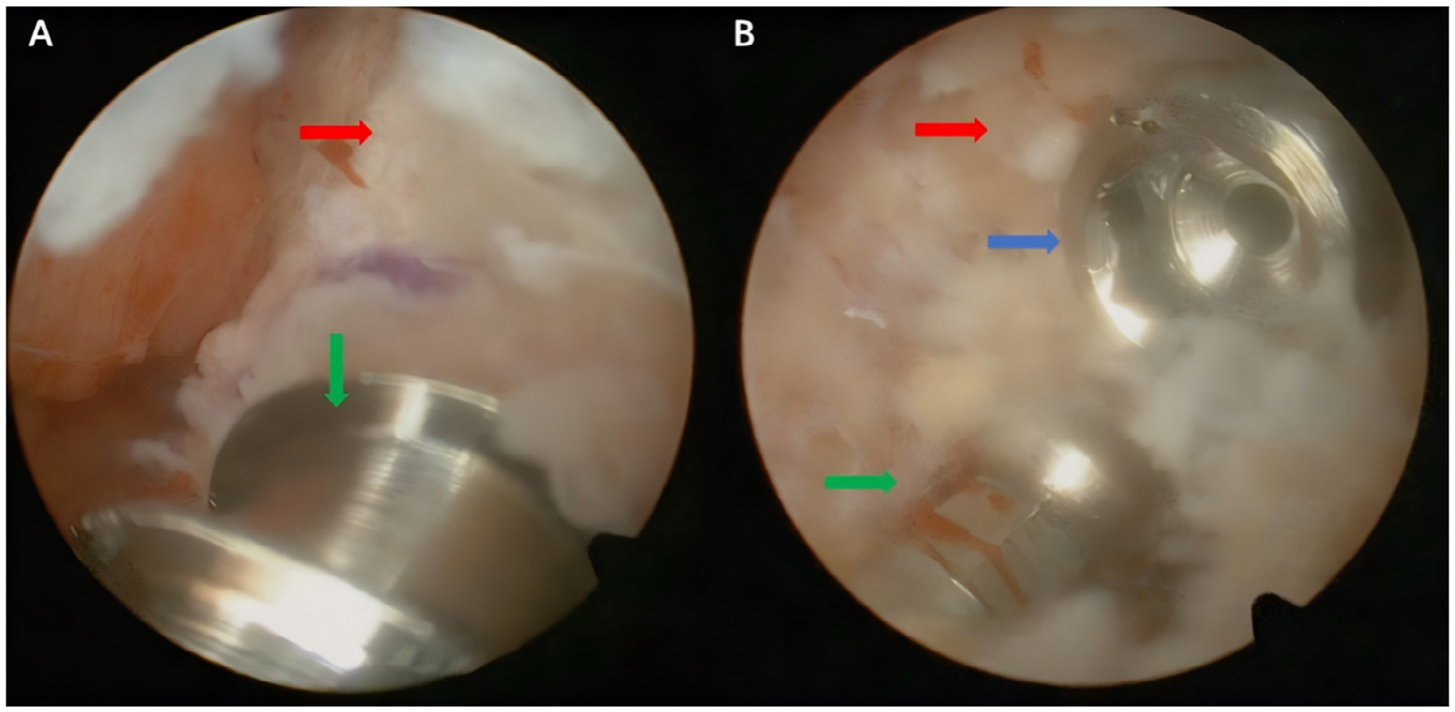
Arthroscopic view of bone block fixation of the right hip in the supine position, viewed from the anterolateral portal. Screws (A,B) are sequentially inserted along the predrilled positions to secure the graft bone block. Red arrow: iliac bone graft; green arrow: first screw; blue arrow: second screw.

**FIGURE 13:**
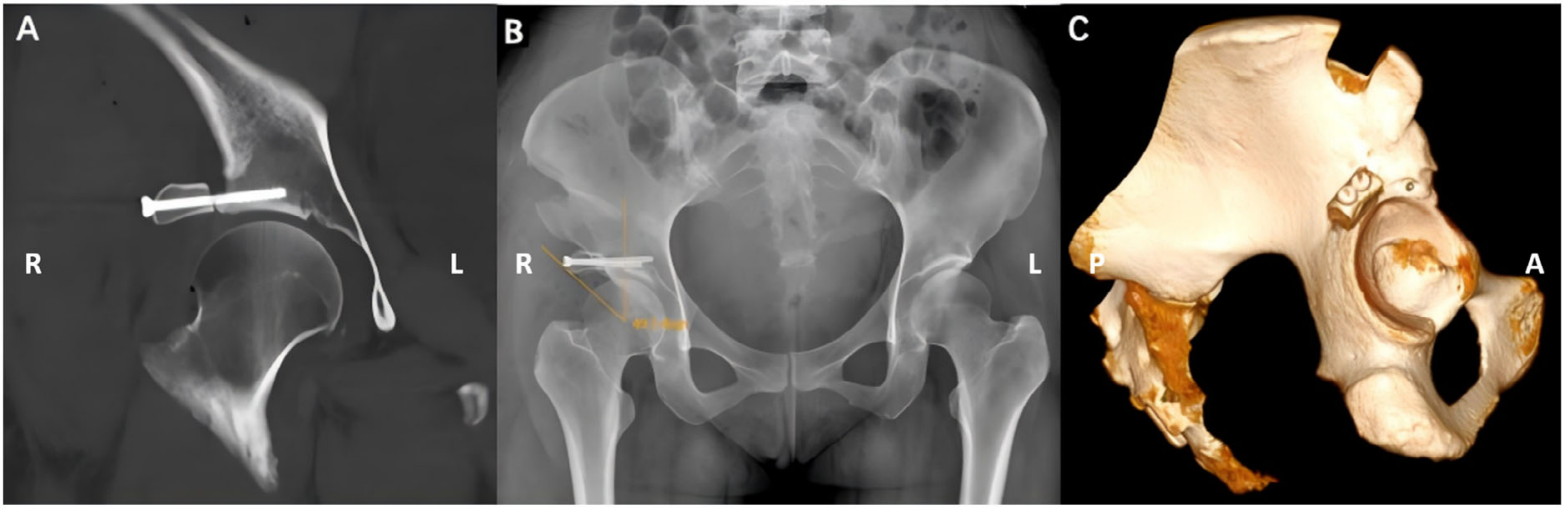
Postoperative imaging of the right hip with the patient in the supine position. (A) Coronal CT scan of the hip joint: The graft bone block matches the curvature of the acetabulum. (B) Radiograph measurement showing a CE angle of 49.1° after fixation. (C) 3D reconstruction of the hip joint CT. (3D, 3‐dimensional; CE, center‐edge; CT, computed tomography.)

An arthroscope is inserted into the space outside the joint capsule. A fluid and tissue management debridement system (DePuy Mitek, Tokyo, Japan) is used to clear the gap to expose the medial edge of the acetabulum. After remove a part of the cortical bone, a Kirschner wire is inserted for drilling and positioning, and C‐arm fluoroscopy is used to determine the pregraft fixation position. A groove approximately 20‐mm long and 2‐mm deep is created using the distal anterolateral accessory approach, similar to that described by Uchida et al.^7^ (Figure 11). The anterolateral approach is enlarged and the graft bone block is inserted along the preinserted Kirschner wire into the acetabular rim. The position of the graft bone block is adjusted according to the fluoroscopic image, to match the curvature of the acetabulum. Sequentially two 4.5 mm screws are inserted, based on the predrilled holes in the graft bone block (Figure 12), and a postoperative imaging examination is immediately performed (Figure 13).

### Postoperative Rehabilitation

For the first month post‐surgery, weight‐bearing on the affected limb is prohibited. During bed rest, hip and knee flexion of up to 90° is allowed, while internal and external rotations and pressure on the affected side are prohibited. After 1 month, weight‐bearing, standing and walking with the aid of crutches are initiated.

## DISCUSSION

In this technique, we combined labral repair, cam and pincer formation, autologous chondrogenesis induced by collagen, and joint capsule suturing in a “five‐in‐one” procedure. To address the issue of insufficient acetabular coverage under arthroscopy, we examined the 4 most common abnormalities related to developmental hip dysplasia. These include labral tears, osseous impingement (cam and pincer deformities), cartilage damage, and joint capsule laxity. The surgical considerations and key points are listed in Table 1.

**TABLE 1:** Surgical Experience and Precautions

Experience	Precautions
After preparing a groove on the lateral margin of the acetabulum, the deep iliac osteotomy surface is combined with it, resulting in a high rate of autologous bone healing	The learning curve for exposing lesions from outside the joint to inside the joint is long
For intra‐articular exposure, we performed lateral joint operations, made punctate incisions, and after determining the position, made a transverse incision along the edge of the labrum to open the joint capsule	Collagen cartilage repair scaffolds are expensive and not available in all countries

A systematic review of the long‐term outcomes of acetabular reorientation surgery indicated that this procedure achieves satisfactory results in treating hip dysplasia in adolescent and adult patients.^9^ Maldonado et al.^10^ described arthroscopic acetabular reorientation using two 3.5 mm cannulated screws, whereas Thaunat et al.^11^ used a single 7.2 mm screw for fixation. In our surgical procedure, the use of 4.0 mm screws achieved stable outcomes.

Cartilage damage in the transition zone between the acetabular cartilage and labrum is associated with increased pain and decreased functional levels 24 months postoperatively.^12^ As the articular cartilage is a tissue with low cellularity, low vasculature, and a limited lymphatic system, its healing capacity is poor, making cartilage injury treatment challenging for orthopaedic physicians.^13^ Cartilage repaired using simple microfracture techniques is usually fibrocartilage rather than hyaline cartilage, and the quality of repair remains uncertain.^14^, ^15^ ACIC is a novel technique used for arthroscopic cartilage repair. It involves injecting a collagen cartilage repair scaffold (COLTRIX CartiRegen) at the site of cartilage damage, providing a regenerative carrier to promote cartilage repair. Ultimately, this results in hyaline‐like cartilage tissue that closely resembles native cartilage.^16^

Our technical advantage lies in offering a method for performing arthroscopic surgery on acetabular dysplasia, which allows for reconstruction of the acetabulum while repairing intra‐articular damage with less invasiveness than periacetabular osteotomy. Additionally, the integration of ACIC technology during surgery promotes healing of autografts and the acetabulum, reducing the risk of postoperative cartilage degeneration. Moreover, for the suturing of the joint capsule, we used the 2 tail lines of the anchor separately to secure the labrum and fold and suture the joint capsule tissue, which helped reduce surgical costs.^8^ However, this technique has certain limitations, mainly owing to its short study duration, lack of long‐term follow‐up, and insufficient large‐sample data, as shown in Table 2.

**TABLE 2:** Pearls and Pitfalls

Pearls	Pitfalls
The tail lines of the anchor are used in 2 different ways: one secures the labrum, and the other sutures the distal joint capsule tissue. This approach not only effectively reduces surgical costs but also minimizes the number of implants required	When developmental hip dysplasia is accompanied by severe osteoarthritis, the reconstructed graft cannot alleviate the patient's symptoms
In one surgery, extra‐articular reconstruction of the acetabulum can simultaneously address intra‐articular lesions, such as labral repair, cam and pincer formation, and cartilage repair	Autologous iliac bone grafting may have some degree of absorption, with a lack of long‐term follow‐up
Through minimally invasive arthroscopy, muscle damage seems to be less, recovery time is fast, postoperative pain is light, intraoperative blood loss is less, and the risk of lateral femoral cutaneous nerve paralysis is low	

## DISCLOSURES

The authors (J.Z., Z.X.) declare the following financial interests/personal relationships which may be considered as potential competing interests: J.Z. reports financial support was provided by Beijing Municipal Administration of Hospitals, Beijing Nova Program, Beijing Jishuitan Hospital Affiliated to Capital Medical University, and Beijing Medical and Health Science and Technology Promotion Center. Z.X. reports financial support was provided by the National Natural Science Foundation of China. The other authors (D.Z., Z.Z., S.Y., D.L., X.Z., C.S.) declare that they have no known competing financial interests or personal relationships that could have appeared to influence the work reported in this article.

## FUNDING

This work was supported by the Beijing Nova Program (http://dx.doi.org/10.13039/501100005090) (NO. 20250484998, Grant Recipient: Jin Zhang), the National Natural Science Foundation of China (http://dx.doi.org/10.13039/501100001809) (NO. 52301301, Grant Recipient: Zhe Xue), the Beijing Selecting and Advance Plan for Medical Science and Technology Innovation Achievements Transformation (NO. YC202301QX0009, Grant Recipient: Jin Zhang), the Beijing Municipal Hospital Administration “Sail” Program Clinical Technology Innovation Project (Medical‐Engineering Integration Incubation Project) (NO. YGLX202513, Grant Recipient: Jin Zhang), and the Talent Training Program of Beijing Jishuitan Hospital Affiliated to Capital Medical University “Discipline Backbone” (2022) (NO. XKGG202210, Grant Recipient: Jin Zhang).
